# Using an action learning methodology to develop skills of health managers: experiences from KwaZulu-Natal, South Africa

**DOI:** 10.1186/s12913-018-3693-6

**Published:** 2018-11-29

**Authors:** Zandile Masango-Muzindutsi, Lyn Haskins, Aurene Wilford, Christiane Horwood

**Affiliations:** Centre for Rural Health, University of KwaZulu-Natal, 4th Floor, George Campbell Building, Howard College Campus, Durban, 4001 South Africa

**Keywords:** Action learning, Health manager, Newborn care, Health workers, Self-efficacy, Resource limited setting, Africa, South Africa

## Abstract

**Background:**

Strong management skills are key to improving performance of health systems. Action learning, a technique to develop management skills, has been used successfully with health managers but not usually among lower level managers or in low and middle-income countries (LMICs).

**Methods:**

This study uses a qualitative approach to explore experiences, successes and challenges of using an action learning approach to improve skills of managers in neonatal units in KwaZulu-Natal (KZN), South Africa. Eight action learning groups were convened with neonatal unit managers from all 39 district hospitals in KZN, each group had 4–6 participants. Meetings were conducted by a facilitator trained in action learning techniques, and groups met a minimum of ten times over a one-year period. After completion of the intervention, 14 in-depth interviews were conducted with purposively selected action learning participants. Data was transcribed and analysed using framework analysis.

**Results:**

Neonatal unit managers found that action learning generated a sense of empowerment in their abilities**,** trust and confidence among participants was nurtured, problem solving and critical thinking skills were developed, and a continuous support system was created. The action learning process led to several positive changes in neonatal units, which enhanced the quality of care for patients. A number of challenges were also identified, mainly relating to administrative issues such as the provision of a skilled facilitator, permission to attend action learning meetings and logistical issues, including transport and other financial implications.

**Conclusions:**

This paper illustrates that action learning can be an effective and practical method to support public health workers to manage their health units despite the challenges associated with the method. Time, energy and financial resources used to facilitate action learning for this cadre of health workers is rewarded by improved skills of managers and better quality of care for patients.

## Background

Strong management skills at all levels have long been identified as key elements to improve performance of health systems, particularly in LMICs (WHO, 2009). However, many health managers are health professionals with no management experience prior to being offered a management position [1]. Effective management and strong leadership are associated with well-functioning facilities, and productive and motivated staff [2, 3]. For these reasons, it is essential that managers in the public health sector be equipped with skills to effectively perform their duties. In South Africa (SA), managers in the public health sector frequently lack skills and training to manage the daily challenges experienced in health facilities, and this has been cited as one of the reasons for poor service delivery in the public health sector [4].

Action learning is one approach that can be used to address the challenge of improving management skills. The ethos of action learning is to ‘take an attitude of enquiry’ based on the principle that experiences generate knowledge. Action learning seeks to facilitate skill development based on integration of knowledge gained from experience and knowledge gained by formal learning, underlined by critical reflection. Action learning is thus rooted in the theory and practice of adult learning [5]. This technique is well-established and has been widely used in several sectors. Action learning can be described as a continuous group-based process of engagement, learning and reflection where a group of peers meet regularly under the guidance of a trained facilitator over an extended time period. In these action learning groups, participants bring real problems to the discussion with the aim of generating innovative and creative ways of dealing with complex problems [6] and facilitating learning through the development of practical solutions that are implemented with planned intent [7]. Sessions are conducted in a socratic manner, such that participants discuss their problems in a constant back and forth questioning and answering process that elicits critical thinking, and dialogue that encourages generation of ideas and clarification of assumptions [6]. Moreover, the process of action learning encourages learners to reflect on and learn from their own experiences and those of their peers [6].

People learn more and better when learning takes place practically than when passively being provided with information [8, 9]. Action learning has been shown to be an effective method of learning and developing leadership skills particularly for senior level managers in developed countries [10]. This may be attributed to it’s less formal nature making it a practical and interactive approach that is directly related to the day-to-day problems that managers face [11]. Although the technique has been mainly used to develop the skills and competencies of senior managers, it is also used among other ranks [12]. Furthermore, although the method has been successfully used in the health sector [13, 14], it has also been implemented in other sectors such as construction [12, 15]. The action learning technique has been shown to improve confidence, resilience, conflict resolution, team work [16, 17], empowerment [18, 19], leadership skills [20], self-efficacy, critical thinking and problem solving skills [13]. All these skills are important for managers in general but more so for those who work in highly stressful environments such as the neonatal unit.

Health workers working in neonatal units in district hospitals work under extremely stressful conditions to care for critically ill infants. In the South African setting, this may be combined with daily challenges such as insufficient equipment and medication, poor transport to the referral centre, as well as shortage and burn-out of staff, making it difficult for middle management to critically develop ways of managing all the problems encountered. Neonatal units in district hospitals are run by generalist doctors and nurses, without any on-site paediatrician, and are often geographically isolated, far from specialist care. Managers of neonatal units are faced with day-to-day problems for which solutions are required. Therefore, it is important to assess whether action learning can be an effective method to improve managers’ skills to address their challenges. This paper evaluates an action learning intervention that was employed to develop the management skills among middle level health managers with direct responsibility for day-to-day management of neonatal units in district hospitals in KZN, South Africa.

## Methods

### Study setting

This qualitative study was conducted in the province of KZN, South Africa. KZN has a population of approximately 10.9 million people [21], with an overall HIV prevalence of 16.9% in 2012 [22] and 40.1% of women attending government clinics for antenatal care tested HIV positive in 2013 [23]. There are 39 district hospitals, ten regional hospitals and one tertiary hospital that provide neonatal care services in the province.

### Sampling

Participants in each action learning group were purposively selected to participate in the evaluation on completion of the action learning process, based on their willingness to share their thoughts and experiences of participating in an action learning group. In-depth interviews were conducted with 14 operational managers or unit mangers of the neonatal units in district hospitals.

### The action learning intervention

#### Facilitator training

The intervention was conducted by three facilitators who received training at a one-day workshop conducted by a facilitator with special skills and experience in action learning. The rationale, principles and technique of action learning were explained and discussed in an interactive manner. The newly trained facilitators were then mentored during their initial group meetings, until they were competent to manage the groups independently. During the training, confidentiality was emphasised as a key factor for participants to pay attention to at all times because of the sensitive nature of the challenges brought the group.

#### Selection of action learning participants

The participants were identified through their respective hospitals. All hospitals providing neonatal services in the province of KZN were included in the intervention and offered the opportunity for the neonatal unit manager to be included in a skills development initiative. Forty-one participants were selected by their facilities to participate in the intervention. Participants were all registered nurses and were included based on being responsible for the day-to-day management of the neonatal unit. All action learning group participants attended a one-day workshop where they were given information about action learning, and the rationale for its use in their work settings, so participants gained a thorough understanding of the requirements expected from them during their participation in the action learning group.

#### Action learning groups

Eight action learning groups were convened with participants from all 11 districts of KZN. Each group consisted of 4–6 participants. Participants were usually from a single district but at times two or three small districts were combined to ensure sufficient participants in the group. Each group had a maximum of 10 sessions and met once per month at a convenient venue.

During the action learning session, group members took turns presenting a problem that they were experiencing at work. The role of the other group members was to listen and critically challenge the presenter to think of their problem in a way that provided possible solutions, and to develop an action plan to implement in their health facility. At the following meeting participants provided feedback on how they had implemented their action plan, and whether the selected strategy had brought about any change in terms of solving their problem. If the participant had elected to implement an alternative action plan, this was also highlighted with the outcomes of this alternative action plan being presented.

### Data collection

A semi-structured interview guide for the evaluation process was developed (see Table 1). This covered topics of interest including participants’ understanding of action learning, how action learning assisted participants in their work settings, and the changes that occurred in the workplace as a result of action learning, challenges in the implementation of action plans and perceptions about facilitation of the action learning process. A researcher trained in conducting in-depth interviews, interviewed the action learning participants. Interviews were conducted after the last action learning session at local conference venues, participants’ health facilities or at the researchers’ organisation. All venues used allowed for privacy to be maintained during the interview.

**Table 1:** Focus group discussion guide

1. What do you understand by the term ‘action learning’? Explain	
2. Describe the experiences you have had of the action learning process Probe: Experiences of attending the groups? Experiences of implementing plans in the workplace?	
3. Are there any ways in which action learning has assisted you in your work? Explain?	
4. Is there anything that has changed in how you do your work as a result of action learning? Explain.	
5. How has the action learning process affected your relationship with your colleagues? Explain	
6. Have there been any challenges in implementing the action learning process in your work? Explain? Probe: Is there any way that the action learning process could be changed or improved?	
7. What can you say about the relationships with the other members of your action learning group? What can you say about the relationship with the facilitator of your action learning group?	
8. What are the three main things that you have learned from participating in action learning?	

### Data analysis

Thematic analysis was used as a method of analysis while NVivo version 11, was used to organise data for ease of reference. Braun and Clarke [24] describe thematic analysis as ‘a method for identifying, analysing and reporting patterns (themes) within data’ (p. 79). It allows the researcher to organise data into codes and themes from both an inductive and deductive approach. Thematic analysis follows a series of steps including familiarising yourself with your data, generating initial codes, searching for themes, reviewing themes, defining and naming themes and producing the report [24].

During the analysis phase, two researchers from the research study team independently coded and analysed the transcripts. The two researchers coded a subset of transcripts independently and had regular meetings to discuss any discrepancies in their coding, any differences were discussed until consensus was reached. Findings were then discussed with the research team, to ensure accurate interpretation of the data. Inter-coder reliability tests were also performed on NVivo to ensure the reliability and rigour of the analysis of data.

## Results

14 interviews with operational managers and unit managers were conducted between 2014 and 2015 with selected action learning group participants. All participants were female and were registered (professional) nurses. Table 2 indicates the number of participants interviewed per action learning group.

**Table 2:** Participants interviewed for action learning evaluation

District/ Action Learning group	District designation	No. of participants interviewed
Ethekweni	Urban	2
Ilembe	Rural	1
Ugu	Rural	1
Umkhanyakude	Rural	3
Zululand	Rural	1
Umzinyathi / Amajuba	Rural	3
Uthungulu	Rural	1
Uthukela / Umgungundlovu / Harry Gwala	Rural	2

A number of themes emerged from the interviews including: benefits gained from action learning within the context of their work; changes that occurred in their respective facilities as a result of implementing action plans; challenges they experienced; and recommendations on how the action learning sessions may be improved.

### Benefits of action learning

Several participants expressed that action learning was empowering and gave them confidence, assertiveness and a sense of independence. The group meetings empowered participants to be proactive in finding solutions to challenges in their units and made them more confident to share ideas and mobilise other staff members to support new approaches to solving problems. Participants also stated that action learning empowered them to approach their managers with ideas on how to improve quality of care for patients within their units.

All the participants mentioned that they had developed confidence through participating in the action learning sessions and that they applied the competencies in their work.

*No, before that I didn’t have that confidence, you know, but after I’ve started this action learning I became so grateful, you know, and have that confidence that really I’m going…Yah, I’m going to win…* (Group 4, Rural District, Operational Manager, Interview 2).

Another participant also shared similar sentiments about the benefits of action learning in relation to her work.

*It made me confident…assertive and confident, that process actually makes you stronger in the whole process…* (Group 1, Urban District, Operational Manager, Interview 1).

Trust among the group members was another key feature that was identified by participants. As a result of the similarity of the challenges experienced by participants and their frequent meetings, participants learned to trust one another and developed lasting relationships that continued beyond action learning.

*It’s a slow process, but you learn to develop the trust in a group and that was crucial, to have trust and confidence in a group, because we were people, although working in the same field and nursing the same people we did not know each other, so we had to develop a trust amongst ourselves first.* (Group 1, Urban District, Operational Manager, Interview 1).

Action learning provided a platform for participants to develop problem-solving skills that they could then practically apply to managing their work more effectively. Participants also mentioned that the iterative socractic model of interrogating a challenge brought to the group, improved their critical thinking skills which were applied to their work challenges in order to develop relevant solutions. Moreover, these skills encouraged the participants to take initiative in their work because they did not rely on senior managers to propose ideas on how to tackle challenges.

*The questions of what the problem statement is, what have I done, what could I have done differently, what did I do instead and what do I need to do in the future, you know – things like that, those were beneficial questions. And they are applicable to every situation, it’s made me look at each problem or challenge that we have to use the same principal for questions for resolving issues.* (Group 3, Rural District, Operational Manager).

The participants further mentioned that action learning provided a support system. The nature of participants’ work meant that they often encounter similar problems in their respective work environments, so they could use similar ideas and solutions in their own health facilities. In addition, the sense of camaraderie that developed in the groups continued after the sessions had ended and participants kept in contact with one another regarding progress on their action plans. The relationships developed provided platforms for participants to communicate regarding challenges that they faced after the intervention ended. This included support with resources such as borrowing supplies when one of the facilities had challenges with ordering.

*I did not even know who my colleagues were in the district* per se*, we maybe met at a district meeting once a quarter or whatever, but we never met as a group where we could trust one another and pick up the phone and phone and say – I have a problem with this baby what do I do?* (Group 1, Urban District, Operational Manager, Interview 1).

### Changes that occurred as a result of action learning

As part of the action learning sessions, each participant was required to present one or more problems that they experienced in their work place. Participants shared a number of problems and some of the solutions implemented from the ideas generated during the action learning sessions. Problems ranged from staffing issues, daily administrative functioning of units, equipment issues, clinical care, infrastructure issues and research and communtiy education. While some action plans were ongoing and some of the problems experienced were not entirely resolved; a number of the participants were able to successfully resolve their challenges with the assistance of action learning. A summary and examples of some of the problems identified during the action learning sessions is shown in Table 3.

**Table 3:** Problems identified during action learning sessions

**Staffing**	
How can I solve the problem of unethical behaviour of nursing staff in the neonatal nursery?	
How can I reduce the high rate of absenteeism of staff in our maternity?	
How can I ensure that the maternity department including nursery is well staffed day and night?	
**Administration and day-to-day running of the neonatal nursery**	
How can I ensure that the kitchen is utilised for dishing out food instead of dishing in the middle of the ward?	
How can I ensure that nursery records are stored safely and accessible?	
How can I prevent contaminated expressed breast milk being left in the fridge?	
**Equipment**	
How can I deal with incubators that are not working in the neonatal nursery?	
How can I ensure that I get proper equipment for administering and monitoring intravenous fluids in the nursery?	
How can I get a − 40 degrees’ fridge (for vaccines)?	
**Infrastructure issues**	
How can I ensure that there is a functioning heating system in our units to prevent hypothermia in our nursery?	
How can I motivate for a boarder mother facility?	
**Clinical care**	
How can I initiate nasal CPAP in the neonatal nursery?	
How can I prevent the increase in caesarean sections in the hospital?	
How can I improve resuscitation skills in maternity?	
How can I get doctors to comply with the neonatal protocols?	
How can I ensure the correct people are on the selection committee for choosing staff members who will go for training?	
**Research and community education**	
How can I explore the issue of babies born outside of the hospital?	
How can I influence the community and traditional healers to understand the dangers associated with the use of traditional medicines during pregnancy?	
How can I explore possibilities for external funding for the boarder mothers facility?	

Additionally, some of the participants success stories of the positive changes that occurred in their units are included as case studies in Tables 4 and 5. The case study shown in Table 4 illustrates how one of the participants used skills gained from action learning to take the initiative to coordinate the compilation of data in her unit. The case study also illustrates how the participant felt empowered to understand that the issue required a number of different personnel in order to be resolved, and that other responsible managers could also assist. The implementation of her action plan not only resulted in her resolving the problem of data management, but also resulted in the development of policies on data management in her unit.

**Table 4:** A lesson in data management

*Participant:..It was just one problem that I had from the beginning that took a while to resolve, it had something to do with data management.*	
***Interviewer: Yes.***	
*Participant: So bringing that problem (to action learning), I think most of the managers told me towards the end (of the action learning meetings) that they all have…the same problem.*	
*So with this process it was like – it was a huge task and we thought that it was just my responsibility. Data management is my responsibility but throughout the [action learning] process we realized that a very small portion of the data management was our responsibility. It was all the other key factors that were put in place to make sure that the [data] management was right, that fell off. We were able to identify these problems.*	
***Interviewer: In the Action Learning?***	
*Participant: Yes, in the Action Learning, with their guidance in the whole group I took it back to my institution to call the* ***FIO (Facility Information Officer)*** *and ask(ed) her – what happened with the data and this is the process, I developed a flow chart. It’s only when the flow chart went up that we realized ok, “these are where the problems are”, physically looking at it and seeing the problems, these were where the problems were, so we took it back, so much so that the* ***FIO*** *developed a policy.*	
***Interviewer: Wow!***	
*Participant: She developed a policy on data management and, well, I had to develop one for my ward as such, which is a draft policy but hers actually went through the whole [Department of Health] system.*	
***Interviewer: Shew!***	
*Participant: She developed a policy on (data) management, which we did not have.*	
(Group 1, Urban District, Operational Manager, Interview 1)

**Table 5:** Improving absenteeism rates

*Participant: I have (had) a problem of absenteeism and eh…*	
***Interviewer: People just not coming to their shifts? Coming to work?***	
*Participant: Yes*	
***Interviewer: Is that what you mean by absenteeism?***	
*Participant: People were not coming to work, they will produce sick notes*	
***Interviewer: Ok***	
*Participant: Yes you find that someone has been absent from work for 8 days in a month, eh others would have (a) pattern of absenteeism. You find that if she’s working during this weekend she won’t come, then the following weekend she will be off. The next one she’s supposed to come, she won’t come again and she will end up being away for all the weekends in a month. Now in that way, the workload became more for those that are still working, coming to work. Now we had to assess this, sit down, and talk to my Assistant Manager about it. We tried to talk to the staff but it was not that helpful. By coming here (to action learning)…we discussed the problem with eh our facilitator*	
***Interviewer: Carry on***	
*Participant: Uh I was empowered because I had to go back[to work] and review, talk to the staff and we also looked at the doctors that were booking these people [off sick] and we realised that mostly it was the sessional doctors because they do not go to the HOS where they can be seen within the hospital*	
***Interviewer: What is the HOS?***	
*Participant: It’s the…OHS-Occupational Health Services*	
***Interviewer: Oh ok***	
*Participant: They don’t utilise that one, they go outside, then the sessional doctors will book them off. Now we were so troubled that we didn’t know how to…we tried to talk to the people but people…will continue absenting themselves until we decided to refer them to the EAP*	
***Interviewer: What’s that?***	
*Participant: EAP is the Employee Assistance Programme by the Employee Assistance Practitioner. We referred them there, they started to…initially they refused to go there, but because there is a form that you have to fill if you are not willing to see (the) EAP. I told them “if you are not willing, you are free not to go there but you must just sign for me that you are not interested in seeing the EAP”. Then they ended up attending EAP*	
***Interviewer: Ok***	
*Participant: Where they got counselling and all…had to explain what is meant by absenteeism and how…it affect(s) the quality of work; being away from work when you are expected to be there. I saw a great, great improvement after they’ve been to the EAP, eh people are coming to work now though there are those few but the majority is doing so well up to now*	
***Interviewer: And did action learning give you the idea to do that?***	
*Participant: The EAP, in fact the EAP is there in the hospital but it was the idea from this side (action learning group), “Have you seen the labour relations? Have you tried the EAP?” Then I said “no I didn’t” then I was empowered that you better (do that), because you have tried, you have counselled people…maybe refer them to a higher level of counselling then I refer(red) them and now the situation is much, much better* (Group 6, Rural District, Operational Manager, Interview 2)	

### Challenges of action learning

Some participants stated that they experienced challenges with the implemenatation of their action plans in their respective units. The challenges mentioned included not having a platform to present their ideas to senior management, going back and forth between colleagues during the implementation process, and colleagues not being receptive to ideas that brought about change in their units. Ultimately, this created delays in implementations of action plans because of the amount of time it took to get approval to implement proposed ideas.

*There is no boarder mother facility. So … we found a method and a way to do that, I mean I took it back … there was just no … like way to present to management because there was no management. So we tried other methods like approaching the quality manger and things like that, and writing letters…, for me nothing has helped with my major problem it still hanging in the air.* (Group 8, Rural District, Operational Manager, Interview 1).

### Coordination of sessions and support from management

Despite the positive experiences stated by the participants, they also revealed that providing coordination and support for action learning meetings was challenging. Obtaining permission from line managers was highlighted by some partcipants as a challenge, as managers did not always understand their being away from work to attend action learning sessions. Moreover, logistical matters including communication and transport to the meetings was a challenge mentioned by some of the participants. This was especially true for those who worked and stayed in remote areas.

*We had a venue at [name of hospital], we were from different hospitals, so we could not have it in one hospital. I think the challenge for me was travelling to [name of hospital], the distance of travelling and the road, with the road works and things like that.* (Group 1, Urban District, Operational Manager, Interview 1).

As part of the evaluation process, participants were also asked how action learning sessions could be improved or changed. The participants mentioned two main things. One was that action learning be an on-going process, where other colleagues could also join and learn. Another was that top management be part of action learning so that they could understand the challenges that participants brought forward as well as support the need for ongoing action learning for the neonatal managers.

*I think if I can improve action learning I can involve the top level management in this action learning programme like the CEO, the medical manager, all the top people that are involved in the management of our patients in the institution.* (Group 5, Rural District, Operational Manager).

## Discussion

Our findings suggest that action learning can be implemented successfully among middle-level health managers in challenging resource-limited settings, benefiting the individual health worker and our findings suggest that this may have led to improvements in the daily functioning of the neonatal units. Health workers are trained to be competent clinicians, and not managers, so development of management skills is crucial when they take up management positions. Action learning participants reported that they felt empowered as a result of participating in the intervention, and that they gained practical skills to tackle their duties within the workplace. Action learning also helped to cultivate collegial relationships among the participants, who continued to seek advice and assistance from their colleagues after the groups had ended.

Action learning implementation is embedded in a cultural and social context, which determines how it is practiced. The origins of action learning lie in western developed countries, and it is strongly based on Western ideas and assumptions. Although action learning has been implemented in a wide variety of diverse cultural settings, there could be concern about adapting this approach to an African setting with a very different pedagogical tradition. In particular, action learning techniques of challenging participants may be threatening in a context where this is not common practice. This study suggested that trust was an important element of success, which took time to build, but otherwise this approach was well received and accepted, and could be more in line with traditional community based approaches to learning [25].

One of the underlying principles of action learning is that groups should be closed, that is, participants remain the same throughout the process, and discussions conducted in groups should be confidential. Therefore, participants need to be committed to the process by attending all meetings. All these aspects contributed to the development of a trusting environment. Such an environment is required to allow discussion of sensitive aspects of a problem, like conflict with colleagues in the workplace and allows group members to challenge each other. The same sorts of trusting relationships may not be easy to achieve using other approaches, particularly in our context where colleagues frequently work in close-knit, isolated communities, making it challenging to ask for advice or to reveal a lack of skills to direct colleagues. Action learning promotes peer learning between members which can be a powerful and accessible tool. Similarly, Leggatt et al. (2011) also found that a psychologically safe environment is key to enabling participants to freely voice their thoughts in the action learning group.

Additionally, our findings indicated that action learning created a support network for participants such that they built relationships that encouraged peer learning [26], maintained contact and continued to support and advise one another beyond the problems addressed in the group meetings. To the authors’ knowledge, this is a new and interesting finding within the realm of action learning and shows that this method is effective and may be sustainable with participants from different geographical settings. Although one of the requirements for a functional action learning group is to have a trained facilitator, sustainability could be achieved as the group becomes more familiar and confident with the action learning process so that they take over the facilitation of the group themselves. In this way, this type of learning promotes self-direction and independence rather than reliance on experts that are percieved as more knowledgeable [27].

Another critical finding from this study is that participants expressed that participation in the action learning groups gave them a sense of empowerment to tackle challenges. Health workers further developed a sense of resilience and problem-solving as a result of many critical problems that were resolved.

Action learning takes on a socratic approach which involves an iterative and reflective process of one’s challenges and the possible solutions. By employing this method, action learning allowed the participants to think critically about issues and come up with solutions on their own. These are generic skills that can be applied to any work situation and can be anticipated not only to lead to changes in the work place during implementation of the action learning groups but to provide skills that will promote personal development of participants, allowing them to be more effective beyond the lifetime of the group.

For any skills development intervention to be successful, particularly when participants are working in isolated areas, considerable time and resources have to be spent, and using action learning as a method to develop skills for health managers is no different. Furthermore, the development of management competencies is complex but the action learning approach may provide immediate benefits in terms of addressing practical workplace challenges. However, actions could be taken to reduce costs, for example meetings could be held at internal venues and conducted at the same time as other routine district management meetings. Using action learning more broadly could allow for different cadres of health workers to use the method, and for the method to be used as a generic process that can be applied to resolving various challenges within the health system. Action learning faciltators could be trained among Department of Health staff but would have to be carefully selected to maintain the trusting relationships within the action learning groups. Implementation of action learning could contribute towards shifting some of the practices and perceptions related to training programmes so that these are considered an integral part of health workers’ jobs rather than being regarded as external, unnecessary and time-consuming activities.

Participants expressed lack of support from senior management as one of the challenges they encountered with regards to attendance of sessions, especially during the initial stages of the intervention. Participants also stated that they needed the support of their managers for the problems identified during action learning to be resolved. SA Nelson and RK Yeo [12] state that ‘the success of action learning programmes will depend heavily on senior management support and the preparedness to commit to longer term learning programmes rather than two-day “sheep dip” training sessions, which accomplish very little at great expense’ (p. 305). This is essential if quality of care is to be improved in such a way that staff are competent and have the support required to provide good standards of care.

Orientation of senior management to action learning as a skills-development tool ahead of implementation could address many concerns that senior managers may have regarding action learning and resolve some of the logistical barriers identified, like obtaining permission to attend the action learning sessions. An additional role for action learning facilitators, if trained within the Department of Health, to improve success and sustainability could be to establish the principles of action learning in each site, improve linkages and communication with senior managers, and advocate for support for action learning at all levels of the health system. Additionally, this could be used as an opportunity to advocate for senior managers to use action learning themselves especially considering its successful use among this group of managers [9]. Furthermore, a reporting tool could be developed in order to track participants’ progress and for senior management to have a good sense of action learning outcomes.

### Limitations

Our study attempted to illustrate how action learning can be used as a method to develop skills among middle managers in resource-limited health facilities. The results of our study are not intended to be generalizable, however through rich accounts, they provide important insights into the outcomes of action learning in our context. Our intervention had limitations in terms of sustainability because it was conducted by external facilitators at venues that were provided. Further research could look at ways of improving sustainability by increasing involvement of senior managers, using internal Department of Health facilitators and promoting the eventual independence of the action learning groups.

## Conclusion

This paper demonstrated that action learning is an effective and practical method for public health workers aiming to manage their health units despite the challenges they face. Health facilities require cadre with effective management skills for optimal service delivery in health care systems. Investing in training of middle managers to acquire necessary management skills is therefore inevitable and feasible, and accessible interventions need to be implemented to provide skills to this group of managers. Action learning could provide the solution but it requires allocation of administrative, logistical and financial resources in order for the process to yield positive outcomes for development of health workers and improvement of service delivery to public health care facility users.

## Abbreviations

KINC: KwaZulu-Natal Initiative for Newborn Care
KZN: KwaZulu-Natal
LMICs: Low and middle-income countries
SA: South Africa
WHO: World Health Organization
